# Model Organisms Facilitate Rare Disease Diagnosis and Therapeutic Research

**DOI:** 10.1534/genetics.117.203067

**Published:** 2017-08-31

**Authors:** Michael F. Wangler, Shinya Yamamoto, Hsiao-Tuan Chao, Jennifer E. Posey, Monte Westerfield, John Postlethwait, Members of the Undiagnosed Diseases Network (UDN), Philip Hieter, Kym M. Boycott, Philippe M. Campeau, Hugo J. Bellen

**Affiliations:** * Department of Molecular and Human Genetics, Baylor College of Medicine (BCM), Houston, Texas 77030; † Department of Pediatrics, Baylor College of Medicine (BCM), Houston, Texas 77030; ‡ Jan and Dan Duncan Neurological Research Institute, Texas Children’s Hospital, Houston, Texas 77030; § Program in Developmental Biology, Baylor College of Medicine (BCM), Houston, Texas 77030; ** Department of Neuroscience, Baylor College of Medicine (BCM), Houston, Texas 77030; †† Department of Pediatrics, Section of Child Neurology, Baylor College of Medicine (BCM), Houston, Texas 77030; ‡‡ Institute of Neuroscience, University of Oregon, Eugene, Oregon 97403; §§ Michael Smith Laboratories, University of British Columbia, Vancouver, British Columbia V6T 1Z4C, Canada; *** Children’s Hospital of Eastern Ontario Research Institute, University of Ottawa, Ontario K1H 8L1, Canada; ††† Department of Pediatrics, University of Montreal, Quebec H3T 1C5, Canada; ‡‡‡ Howard Hughes Medical Institute, Baylor College of Medicine (BCM), Houston, Texas 77030

**Keywords:** functional genomics, *Drosophila*, zebrafish, human, genetic diseases, whole-exome sequencing, diagnostics

## Abstract

Efforts to identify the genetic underpinnings of rare undiagnosed diseases increasingly involve the use of next-generation sequencing and comparative genomic hybridization methods. These efforts are limited by a lack of knowledge regarding gene function, and an inability to predict the impact of genetic variation on the encoded protein function. Diagnostic challenges posed by undiagnosed diseases have solutions in model organism research, which provides a wealth of detailed biological information. Model organism geneticists are by necessity experts in particular genes, gene families, specific organs, and biological functions. Here, we review the current state of research into undiagnosed diseases, highlighting large efforts in North America and internationally, including the Undiagnosed Diseases Network (UDN) (Supplemental Material, File S1) and UDN International (UDNI), the Centers for Mendelian Genomics (CMG), and the Canadian Rare Diseases Models and Mechanisms Network (RDMM). We discuss how merging human genetics with model organism research guides experimental studies to solve these medical mysteries, gain new insights into disease pathogenesis, and uncover new therapeutic strategies.

DISEASE diagnosis, one of the primary goals of medicine, is a process to discover the origin or nature of disease in a particular patient. Without an accurate diagnosis, we can neither identify the cause nor design an effective treatment strategy to suppress or ameliorate the condition. Many patients with rare conditions undergo a “diagnostic odyssey,” a long and frustrating journey to obtain an accurate diagnosis. Several organizations have estimated that ∼80% of these undiagnosed diseases have a genetic origin (IOM 2010).

Advanced technology is increasingly important for diagnosis and has been transformative for rare Mendelian disorders (Gonzaga-Jauregui *et al.* 2012). Whole-exome sequencing (WES) has emerged as an effective diagnostic tool for patients who have undergone comparative genomic hybridization methods to rule out chromosomal abnormalities (Need *et al.* 2012; Yang *et al.* 2013, 2014; Lee *et al.* 2014; Retterer *et al.* 2016). However, sequencing also uncovers many rare variants for which the functional impact is not known (Coventry *et al.* 2010; Lupski *et al.* 2011), and interpretation can be difficult as healthy individuals often carry alleles that would be deleterious or lethal when homozygous (Gao *et al.* 2015). Model organism studies can be a pivotal resource for understanding these variants (Yamamoto *et al.* 2014). In many cases, studying genes simultaneously in humans and model organisms provides additional insight into the cause of a particular disease, thereby contributing to an understanding of the pathogenic processes.

Studying human genomic variants in model organisms is facilitated by collaboration between clinicians and model organism researchers (Hieter and Boycott 2014; McGurk *et al.* 2015; Bonini and Berger 2017; Chao *et al.* 2017b; Manolio *et al.* 2017). We highlighted the importance of model organisms, particularly the fruit fly *Drosophila melanogaster*, in the context of human biology (Wangler *et al.* 2015), and described how model organism researchers are becoming integral to disease research. The questions we raised about the future of model organism research, and subsequent discussion at The Allied Genetics Conference in 2016, which included the Director of the National Institutes of Health (NIH), made clear the priority that model organism research should have in biomedical research.

In this article, we show how model organism research can be integral to unraveling the molecular mechanisms of disease. We begin this discussion with illustrative examples that document the value model organisms have brought to human disease research, including Mendelian diseases and cancer. We highlight three approaches for engaging model organism laboratories with the expanding list of human genes and variants requiring functional annotation. We primarily focus on two model organisms—*Drosophila* and zebrafish (Figure 1)—and highlight large-scale efforts in the United States (US) and Canada that integrate model organisms into human disease diagnosis and therapy development. Projects such as the Undiagnosed Diseases Network (UDN) with its centralized Model Organisms Screening Center (MOSC) (Gahl *et al.* 2016), the Centers for Mendelian Genomics (CMG) (Chong *et al.* 2015) and the Canadian Rare Diseases Models and Mechanisms Network (RDMM) provide exciting opportunities for model organism researchers, human geneticists, and clinicians to collaborate to determine the potential pathogenicity of disease-linked rare variants, gain deeper pathophysiological understanding of diseases, and discover potential treatments and therapies for patients and families. Lessons learned from these complementary projects will likely lead to the establishment of an efficient framework for facilitating the diagnosis and study of rare diseases.

**Figure 1:**
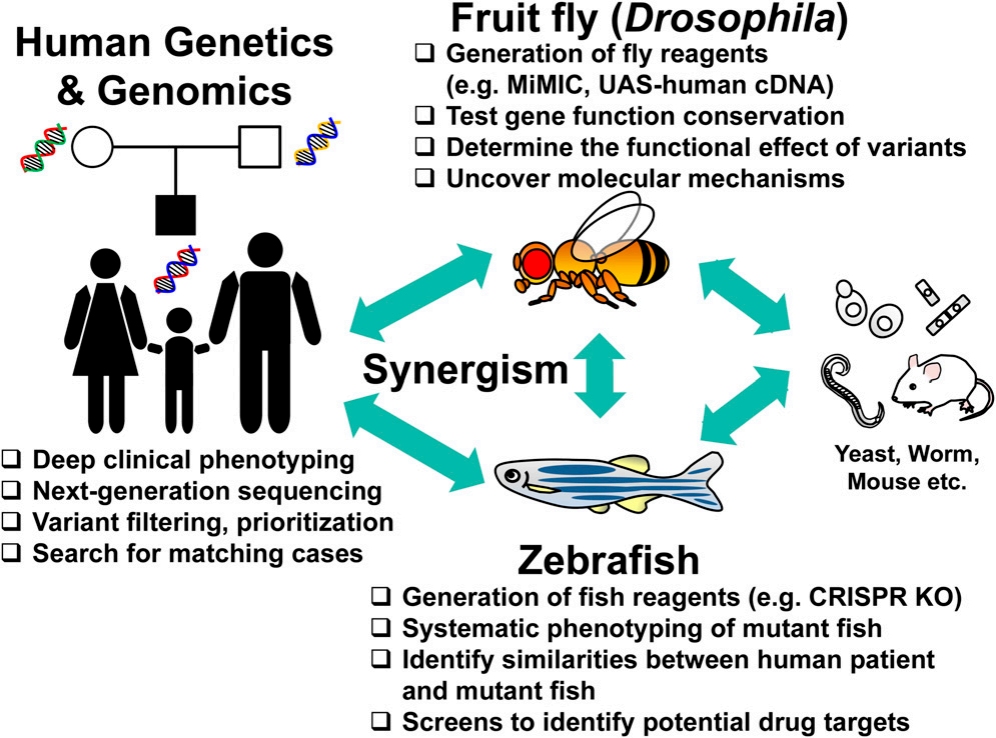
Collaborations among clinicians, human geneticists and model organism researchers facilitate diagnosis and studies of undiagnosed conditions. Candidate causative genes and variants identified from a patient with an undiagnosed disease can be explored in a number of genetic model organisms. Using state-of-the-art genome engineering technologies in these model systems, one can assess whether the variants of interest lead to functional consequences *in vivo*, and obtain phenotypic information that may directly or indirectly relate to the patient’s condition. Integration of biological information from multiple species can be complementary or/and confirmatory.

## Evolutionary Conservation of Genes and Pathways: Orthologs and Phenologs

Biologists often focus on differences among organisms. Indeed, the diversity of life is staggering. Our first impulse is to assume that this diversity arises from different developmental mechanisms, and that these processes must rely on different genes, signaling pathways, and molecular events. For example, it is difficult to comprehend that a fruit fly or a fish could share many genes and signaling pathways with humans. They differ in so many morphological features, and their physiology seems so different, that we tend to imagine there are no or very few commonalities. It was therefore surprising when it became apparent in the 1980s and 1990s that numerous genes are conserved across species and are used repeatedly in many different contexts.

In the past 25 years it became apparent that many biological processes are evolutionarily conserved, and that discoveries in yeast, worms, and flies have a direct relevance to vertebrate and human biology. The 2016 Nobel Prize in Physiology or Medicine was awarded to Yoshinori Ohsumi for his pioneering genetic studies in yeast that led to the discovery and mechanistic understanding of autophagy, a fundamental process involved in cancer, neurological disorders, and other diseases (Takeshige *et al.* 1992; Tsukada and Ohsumi 1993; Mizushima *et al.* 1998). Similar major discoveries with yeast include the identification of core mediators of the cell cycle (Hartwell *et al.* 1970, 1974; Nurse and Bissett 1981; Balter and Vogel 2001) and mechanistic understanding of vesicle trafficking (Novick *et al.* 1980) (2001 and 2013 Nobel Prizes, respectively). Other insights arose from the study of invertebrate animals, including the discovery of molecular mechanisms of learning and memory in the sea slug *Aplysia* (Kandel 2001) (2000 Nobel Prize), RNA interference (Fire *et al.* 1998) and apoptotic pathways (Ellis and Horvitz 1986) in the nematode worm *Caenorhabditis elegans* (2006 and 2002 Nobel Prizes, respectively), and identification of conserved developmental genes and pathways (Lewis 1978; Nusslein-Volhard and Wieschaus 1980) and innate immunity mechanisms (Lemaitre *et al.* 1996) in *Drosophila* (1995 and 2011 Nobel Prizes, respectively) had major impacts on biomedical research. Basic knowledge obtained from model organism research is now being translated to mammalian contexts with remarkable speed. For example, control of organ growth and size was shown to be regulated by genes of the Hippo signaling pathway in flies in 2003 (Halder and Johnson 2011). It took only a few years to show that this pathway is conserved in humans and that it plays a critical role in organ growth, cancer, and regeneration (Saucedo and Edgar 2007).

Conservation of genes and processes extends to numerous physiological pathways. For example, genes encoding proteins that play important roles in synaptic transmission, action potential propagation, and many other forms of neuronal communication or muscle function are highly conserved and play similar roles in worms, flies, and mammals (Bellen *et al.* 2010). Similarly, the following sections illustrate elegant examples of what can be learned about human biology from invertebrates and fish. Obviously, not all genes are evolutionarily conserved from invertebrates to human; ∼35% of human genes have no obvious orthologs in flies (Wangler *et al.* 2015), but can nevertheless be studied in vertebrates like zebrafish and mice. Indeed, zebrafish allow more robust phenotypic comparisons than flies for organs such as the heart, gut, liver, pancreas, and kidney, and have emerged as a key model system to study processes such as hematopoiesis and neural crest development (Boatman *et al.* 2013; Mayor and Theveneau 2013).

One consideration when selecting an organism for disease studies is its overall genomic architecture. After the divergence of the human and fly lineages, two rounds of whole genome duplication likely occurred in the last common ancestor to all vertebrates (Holland *et al.* 1994; Dehal and Boore 2005). As a consequence, humans often have two to four co-orthologs of individual fly genes: for example, four *HOX* gene clusters in humans *vs.* one in flies. According to the principle of subfunctionalization (Force *et al.* 1999), the human co-orthologs likely share among them ancestral functions represented by the single-copy fly genes. Likewise, teleosts (majority of bony fish) experienced an additional genome duplication that provided zebrafish with two copies of ∼25% of human genes (Howe *et al.* 2013). The direct orthology of zebrafish genes to human orthologs is usually unambiguous (Braasch *et al.* 2016), which facilitates connection of zebrafish experimental findings to human biology.

Flies and fish have tissues and organs that are specific to their phyla. One may wonder if wings of flies or dorsal fins in fish can teach us something that is directly relevant to human development or physiology. Mutations that affect the development of wings in flies pioneered the characterization of key developmental pathways such as the Notch (mutants have a “notch” in the wing due to loss of distal wing tissue) and Wingless (Wnt) signaling pathways. Interestingly, pathogenic variants in many genes in the Wnt pathway in humans cause Robinow syndrome—a severe skeletal dysplasia with short stature, short limbs and digits, dysmorphic facial features, and a variety of other problems including cardiac anomalies (MIM# 180700, 268310, 616331, and 616894). Pathogenic variants in *WNT5A* (a homolog of *Drosophila Wnt5*) (Person *et al.* 2010), its receptor *ROR2* (Afzal *et al.* 2000; van Bokhoven *et al.* 2000), as well as mutations in signal mediators *DVL1* (Bunn *et al.* 2015; White *et al.* 2015) and *DVL3* (White *et al.* 2016) (homologs of the fly *dishevelled* gene) cause Robinow syndrome. Thus, although flies do not have an endoskeleton like in vertebrates, conserved genetic machinery that regulates wing patterning in *Drosophila* is responsible for skeletal and craniofacial defects in humans. This parsimony arises because evolutionarily conserved molecular pathways are reused in different cellular contexts and tissues.

These observations led to the concept of “phenologs” or “orthologous phenotypes” (McGary *et al.* 2011). Orthologous genes often cause different phenotypes in different species, yet the proteins encoded by these genes have similar molecular functions. To discover phenologs, one can cluster genes known to cause a human disease and determine the phenotypes caused by mutations in the corresponding genes in model organisms. For example, human genes associated with breast cancer overlap with *C. elegans* genes that mutate to cause a high frequency of male offspring. While the phenotypes in humans (breast cancer) and worms (increased male offspring) do not appear related, they derive from common underlying genetic defects (subset of genes involved in DNA damage response). Once these nonobvious relationships are documented, then additional genetic links can be made between models and humans (McGary *et al.* 2011). For genes that are evolutionarily conserved, simple model organisms with short generation times and amenable to inexpensive and efficient genetic manipulations can yield rapid insights into basic biological functions through detailed *in vivo* studies (Lehner 2013).

Finally, when using one species to understand another, a key experiment is to determine how interchangeable genes are between species if one wishes to assess whether a human variant may be pathogenic. Several studies performed in yeast indicate that many yeast genes (33–47%, depending on the study and assay) can be replaced by their human counterparts (Hamza *et al.* 2015; Kachroo *et al.* 2015). Many human genes can also significantly or fully rescue fly mutant phenotypes, allowing tests of the effects of human variants on protein function *in vivo*. Although this is based on a biased sample of ∼25 *Drosophila* genes, it suggests that this approach can be productive. One of the most spectacular examples of a cross-species gene rescue is the *atonal* gene, associated with deafness, blindness, and loss of proprioception in flies as well as in mammals (Jarman *et al.* 1993). The fly *atonal* gene fully rescues loss of the mouse *Atoh1*gene (Wang *et al.* 2002). Similarly, the mouse *Atoh1* gene rescues almost all phenotypes associated with the loss of *atonal* in flies (Wang *et al.* 2002). These results are remarkable considering that *atonal* and *Atoh1* share only a short stretch of sequence similarity in the DNA binding domain, and most orthology prediction programs do not identify these genes as strong ortholog candidates. We anticipate that, in many cases, testing human genes in model organisms will provide an experimentally productive platform for analysis of gene function, variant pathogenicity, and disease pathogenesis, even when tested in very different biological contexts.

## Model Organism Studies Can Facilitate Diagnosis and Treatment

### Drosophila, TRP channels and a spectrum of Mendelian disorders

One of the earliest examples of a *Drosophila* mutant that informed studies of an extensive gene family implicated in numerous human disorders comes from the fly *transient receptor potential* (*trp*) gene. Studies in the 1960s identified a nonphototaxic fly mutant with a distinct electroretinogram phenotype (Cosens and Manning 1969). Subsequent studies revealed that the affected gene encodes a pore-forming cation channel that is the founding member of a large, diverse family of evolutionarily conserved proteins known as TRP channels (Montell 2005). TRP channel family members have a weak voltage-sensing transmembrane domain, a selectivity filter, and diverse N- and C-terminal domains that provide versatile activation mechanisms. TRP family members encode unique channels that respond to light, sound, chemicals, temperature, pressure, or tactile stimuli, and can integrate multiple signals. The human genome contains 28 TRP channel family members (Gray *et al.* 2015), 11 of which are implicated in Mendelian disorders. These disorders have diverse clinical presentations, different inheritance patterns, and affect distinct tissues (Table 1). Insights from *Drosophila* led to an understanding of TRP channel function, which laid the groundwork for defining the cause of these disorders as resulting from gain-of-function (GOF), loss-of-function (LOF) or modulation (alteration)-of-function mechanisms. The most extreme disease is caused by *TRPV4* (Dai *et al.* 2010)—a gene that underlies a broad spectrum of skeletal and nervous system phenotypes. An autosomal dominant condition *brachyolmias type 3* (MIM# 113500), which is characterized by short trunk, short stature, and scoliosis, as well as a series of more severe skeletal dysplasias, is due to GOF missense mutations in *TRPV4* that causes the channels to be activated by stimuli to which they would not normally respond (Nishimura *et al.* 2012). Other variants in *TRPV4* cause *congenital distal spinal muscular atrophy* (MIM# 600175) and *Charcot-Marie-Tooth disease type 2C* (MIM# 606071) (Deng *et al.* 2010; Landoure *et al.* 2010; Nilius and Owsianik 2010). Heterozygous mutations leading to these neurological phenotypes appear to have a complex impact on channel function and appear as GOF or LOF in different assays (Auer-Grumbach *et al.* 2010; Deng *et al.* 2010; Landoure *et al.* 2010). These functional insights would likely be identified more slowly without the mechanistic understanding of the channels provided by studies in *Drosophila*.

**Table 1:** Select Mendelian disorders from TRP channel family members

Gene Name	Inheritance	Disorder (MIM#, name)	Affected Tissues	Proposed Genetic Mechanism
*MCOLN1*	AR	#252650, Mucolipidosis IV	Neurologic, ocular, gastrointestinal	LOF
*TRPM1*	AR	#613216, Night blindness, congenital stationary (complete), 1C	Photoreceptors	LOF
*TRPM6*	AR	#602014, Hypomagnesemia 1, intestinal	Gastrointestinal	LOF
*PKD2*	AD	#613095, Polycystic kidney disease 2	Renal	LOF (haploinsufficiency)
*TRPA1*	AD	#615040, Episodic pain syndrome, familial	Peripheral nervous system	GOF
*TRPC3*	AD	#616410, ?Spinocerebellar ataxia 41	Central nervous system	Toxic GOF
*TRPC6*	AD	#603965, Glomerulosclerosis, focal segmental, 2	Renal	GOF
*TRPM4*	AD	#604559, Progressive familial heart block, type IB	Cardiac	GOF
*TRPM7*	AD	#105500, Amyotrophic lateral sclerosis-parkinsonism/dementia complex, susceptibility	Central nervous system	Modulation of function?
*TRPV3*	AD	#616400, ?palmoplantar keratoderma	Skin	GOF
		#614594, Olmsted syndrome		
*TRPV4*	AD	Two spectra of Mendelian disorders- skeletal and neurologic (see text)	Skeletal in one group, neurologic in another group	Complex (see text)

AR, Autosomal recessive; AD, autosomal dominant; LOF, loss of function; GOF, gain of function.

### Zebrafish and melanoma

Research on zebrafish complements research on flies because zebrafish share vertebrate-specific features with human, such as similar organ structures. Zebrafish offer a number of experimental advantages for investigation of human disease mechanisms and therapeutic strategies (Phillips and Westerfield 2014). Chief among these are the ease of genetic manipulation, the ability to replace zebrafish genes with human genes, sensitive phenotypic analyses, and the capacity to conduct high-throughput screens of small molecules for potential therapeutics.

Examples discussed here involve human melanomas, which are genetically diverse cancers. This genetic heterogeneity makes it difficult to discover which gene mutations are primary drivers of oncogenesis, and which are critical modifiers that promote metastatic disease. A valine-to-glutamic acid mutation at position 600 in the human *BRAF* gene is the most frequent mutation driving human melanomas, but mutations in other genes are required for metastasis (Pollock *et al.* 2003). In searches for “second hit” loci in *BRAF^V600E^* melanomas, a region of chromosome 1q21 was identified as a key driver of metastasis, but the presence of >50 genes in the identified interval made it difficult to determine the primary driver (Lin *et al.* 2008). Testing genes in the corresponding zebrafish chromosomal interval revealed a single gene, *SET domain*, *bifurcated 1* (*SETDB1*) that cooperates with *BRAF^V600E^* to drive melanoma formation and growth (Ceol *et al.* 2011). The 1q21 interval was subsequently linked to familial melanoma in human (Macgregor *et al.* 2011), establishing *SETDB1* as a major human melanoma oncogene. A high-throughput chemical screen of these zebrafish with ∼2000 substances identified inhibitors of dihydroorotate dehydrogenase (DHODH), such as the anti-inflammatory drug leflunomide, as suppressors of neural crest development and melanoma formation (White *et al.* 2011). This work in zebrafish led to Phase I/II clinical trials of leflunomide in combination with a previously studied BRAF-inhibitor for treatment of melanoma. The ability to create sensitized zebrafish lines with human genes, coupled with the ability to screen thousands of compounds for their capacity to rescue disease phenotypes, illustrates the power of zebrafish studies for illuminating human disease pathogenesis and revealing novel drug targets.

## Model Organisms and Medical Diagnosis

These examples of fly and fish research illustrate the often under-recognized foundational contributions to human health that model organism research provides. Rapid advances in medical technology have expanded the need to incorporate these insights into the practice of medicine. Next-generation sequencing, particularly WES, transformed the diagnosis of rare disease (Gonzaga-Jauregui *et al.* 2012; Yang *et al.* 2014; Retterer *et al.* 2016). Furthermore, the coupling of genomic data with phenotypes extracted from medical electronic health records enables large-scale analysis of the clinical impact of rare variants (Abul-Husn *et al.* 2016; Dewey *et al.* 2016). However, this new sequencing technology presents challenges. Each personal genome contains hundreds of rare and unique variants that can have dramatic functional significance (Coventry *et al.* 2010), but most are without obvious effect. Their sheer number can make interpretation difficult, leaving the majority of individuals with apparent Mendelian diseases undiagnosed (Need *et al.* 2012; Yang *et al.* 2014; Posey *et al.* 2016; Retterer *et al.* 2016). Merging results from many genomes has significantly increased the power of sequence analysis, but, at the same time, has dramatically increased the need to study the impact of gene variants on protein function. We argued for an important role of model organism research studies in this effort (Bellen and Yamamoto 2015). Here, we present several examples of the early success of this approach.

### Centers for Mendelian Genomics

The study of Mendelian disorders has yielded many significant insights into human biology, starting with the recognition of *alkaptonuria* (MIM# 203500) as an example of Mendel’s laws operating in humans (Garrod 1902, 1923). The availability of WES propelled novel disease gene and variant discovery, leading to in-depth cataloging of the function of thousands of genes in the human genome, and providing insights into the mechanisms of both rare Mendelian disease and more common, complex traits (Albert *et al.* 2007; Levy *et al.* 2007; Wheeler *et al.* 2008; Bainbridge *et al.* 2010; Lupski *et al.* 2010; Blair *et al.* 2013; Wu *et al.* 2015). Nonetheless, the diagnostic rate (% of cases in which a pathogenic mutation in a known disease gene was identified) of clinical WES remains at 25–30% (Yang *et al.* 2013, 2014; Lee *et al.* 2014; Farwell *et al.* 2015; Retterer *et al.* 2016; Eldomery *et al.* 2017; Wenger *et al.* 2017). The diagnostic yield varies depending on age and how the patients were selected, with diagnostic rates as high as 57% in infants in a tertiary pediatric center *vs.* 17.5% for adults (Posey *et al.* 2016; Stark *et al.* 2016). A review of Online Mendelian Inheritance in Man (OMIM) reveals 8487 human disease phenotypes with suspected Mendelian basis, of which 6017 (70.9%) have a known molecular basis.

For the past 6 years, the National Institutes of Health (NIH) has funded several sequencing centers to use next-generation sequencing techniques, primarily whole-exome sequencing, to identify and describe as many Mendelian disorders as possible (Bamshad *et al.* 2012). The first 4 years of funding to three independent CMGs (Baylor Hopkins CMG, University of Washington CMG, and Yale CMG) produced discoveries of 956 genes, including 375 not previously associated with human disease (Chong *et al.* 2015). The overarching approach of the CMGs is to identify individuals, families, and cohorts with conditions that have a high probability of being Mendelian, and to apply WES to identify potentially causative disease genes and variants. The CMGs benefitted significantly from development of several computational tools that enable disease gene and variant identification, phenotype-driven analysis, and identification of clinicians and researchers studying the same gene or phenotype (Table 2). One tool essential for the development of these and other studies is GeneMatcher. By allowing researchers to identify each other based on their interest in a gene, GeneMatcher is an active collaboration tool in human genetics. Importantly, this tool also allows model organism researchers to identify human geneticists and clinicians interested in collaborations (Sobreira *et al.* 2015b).

**Table 2:** Bioinformatic tools and databases developed by the Centers for Mendelian Genomics

Function	Name	Description	Interface for Fly Researchers	References
Variant identification	HMZDelFinder	Identification of homozygous and hemizygous deletions from exome variant data		Gambin *et al.* (2017)
Variant identification	DNMfinder	Identification of *de novo* variants from trio-WES data		Eldomery *et al.* (2017)
Phenotype database/analysis	PhenoDB https://www.mendeliangenomics.org/	Searchable database of clinical phenotypes for individuals and their relatives, phenotypes reported by expert clinicians	Rapid identification of patients with phenotypes of potential interest for further study	Hamosh *et al.* (2013), Sobreira *et al.* (2015a)
Phenotype database/analysis	OMIMExplorer https://omimexplorer.research.bcm.edu/	Prioritization of exome variants based on clinical phenotype data		James *et al.* (2016)
Phenotype database/analysis	Geno2MP http://geno2mp.gs.washington.edu/Geno2MP	Searchable database of genotype and phenotype data	Gene and variant-based searching allows identification of related human phenotypes	Chong *et al.* (2016a)
Gene and/or phenotype matching	GeneMatcher https://genematcher.org/	Identification of collaborators studying the same gene and/or phenotype, functions as a MME node	Identification of physicians and/or scientists who may have study patients with a relevant genotype or phenotype	Sobreira *et al.* (2015a,b)
Gene and/or phenotype matching	MyGene2 http://www.mygene2.org/	Phenotype and genotype database that includes patient-entered data, supports data matching, functions as a MME node	Identification of physicians and/or scientists who may have study patients with a relevant genotype or phenotype	Chong *et al.* (2016b)

The potential power of integrating model organism studies into interrogations of the functions of candidate disease genes and variants identified by the CMG is illustrated by the analysis of human homologs of a set of 153 *Drosophila* genes required for nervous system development or function (Yamamoto *et al.* 2014). For ∼30% of these, a human disease had already been associated with the homologs of the *Drosophila* gene. Interrogation of exome variant data from human homologs of the 153 *Drosophila* genes led to molecular diagnoses in six families. In one example variants in a known disease gene for early onset retinal disease (*CRX*), caused a later onset retinal phenotype not previously seen with *CRX* variants. Such a finding is often referred to as a “phenotypic expansion” of a known disease-linked gene. In another example, a novel disease gene whose biological function was poorly characterized (*ANKLE2*) was discovered to be linked to *autosomal recessive primary microcephaly 16* (MIM# 616681). Within 2 years of the publication of the screen of these 153 fly genes, an additional ∼15% of the genes have become linked to Mendelian diseases. A key insight from this study led to the realization that essential genes in *Drosophila* with more than one human homolog are eight times more likely to be associated with Mendelian diseases, allowing the prioritization of disease candidate genes (Yamamoto *et al.* 2014).

There are now several additional examples of novel disease-gene discoveries by the CMG that benefited from collaborative functional studies in *Drosophila*. These include: *metabolic encephalomyopathic crises*, *recurrent*, *with rhabdomyolysis*, *cardiac arrhythmias*, *and neurodegeneration* (MIM# 616878) and *TANGO2* (Bard *et al.* 2006; Kremer *et al.* 2016; Lalani *et al.* 2016); *cerebellar atrophy*, *visual impairment*, *and psychomotor retardation* (MIM# 616875) and *EMC1* (Harel *et al.* 2016a); *Harel-Yoon syndrome* (MIM# 617183) and *ATAD3A* (Harel *et al.* 2016b); *lissencephaly 6 with microcephaly* (MIM# 616212) and *KATNB1* (Mishra-Gorur *et al.* 2014); *steroid-resistant nephrotic syndrome* (MIM# 615573) and *ADCK4* (Ashraf *et al.* 2013); and *NRD1* and *OGDHL* as novel disease genes associated with nervous system phenotypes (Yamamoto *et al.* 2014; Yoon *et al.* 2017). A key characteristic of these efforts is the bidirectional impact of insights: candidate genes identified from sequencing human patients can be studied with reverse genetics in flies, and orthologs of essential *Drosophila* genes can be examined in human genomic data. These bidirectional approaches provide a synergistic effect (Figure 1) (Wangler *et al.* 2015).

Studies of other model organisms also support novel disease gene discovery efforts. For example, the discovery of an association between variation in *FAT1* and a human phenotype of steroid-resistant nephropathy with neurologic involvement benefitted from studies in zebrafish and mice, demonstrating that loss of Fat1 leads to abnormal podocyte and brain development (Ciani *et al.* 2003; Skouloudaki *et al.* 2009; Gee *et al.* 2016). Both organisms also contributed to the identification of additional genes associated with abnormal brain structure or function, such as *CLP1* in *pontocerebellar hypoplasia* (MIM# 615803) (Karaca *et al.* 2014; Schaffer *et al.* 2014), *KATNB1* in *lissencephaly 6 with microcephaly* (MIM# 616212) (Mishra-Gorur *et al.* 2014), and *FMN2* in *nonsyndromic intellectual disability* (MIM# 616193) (Law *et al.* 2014). Both organisms also proved useful in the study of non-neurologic phenotypes, such as the role of *ZSWIM6* in *acromelic frontonasal dysostosis* (MIM# 603671) (Smith *et al.* 2014), *CAV1*, and *neonatal lipodystrophy* (Garg *et al.* 2015), *WDPCP* (MIM# 217085 and 615992) and *INTU* in *ciliopathy syndromes* (Toriyama *et al.* 2016), and *FOXE* in *thoracic aortic aneurysms and dissections* (Kuang *et al.* 2016).

### Undiagnosed diseases program and network

Patients with a rare disease often remain undiagnosed or misdiagnosed for years (Markello *et al.* 2011). The “diagnostic odyssey” experienced by many of these patients is an emotional and financial burden to patients, families, and the healthcare system. Addressing this problem was part of the motivation for the establishment of the Undiagnosed Diseases Program (UDP), a NIH intramural program led by the NHGRI (National Human Genome Research Institute) (Gahl *et al.* 2012). The UDP model is unique for several reasons. First, the patient is the applicant and the patient’s participation drives the process, which is in contrast to most other clinical studies in which referring physicians are the primary applicants. Second, the study starts with a broad and in-depth clinical evaluation. Third, this model of systematic and tailored clinical phenotyping for single, unique cases is followed by genomic assessments using the latest technologies. By accepting applications from individuals with diverse undiagnosed conditions, sorting through thousands of applications to identify patients with strong objective findings, performing extensive phenotyping and clinical testing on accepted patients at the NIH Clinical Center, and performing WES in the majority of the cases, the UDP is able to provide diagnoses for patients and families who have exhausted traditional diagnostic modalities (Tifft and Adams 2014). Successes include the description of new disease genes, such as *NT5E* in arterial calcifications (St Hilaire *et al.* 2011). The genetic definition of this rare condition implicated adenosine metabolism in more common cases of vascular pathology (Markello *et al.* 2011). In addition, the effort has uncovered cases of known conditions that were missed because of misleading laboratory data or unusual phenotypic features that obscured the diagnosis (Gahl *et al.* 2012), and revealed “phenotypic expansion” for disease genes such as *AFG3L2* (Pierson *et al.* 2011). These efforts are valuable for the medical care of patients, and provide a better understanding of the unanticipated functions of genes.

**Figure 2:**
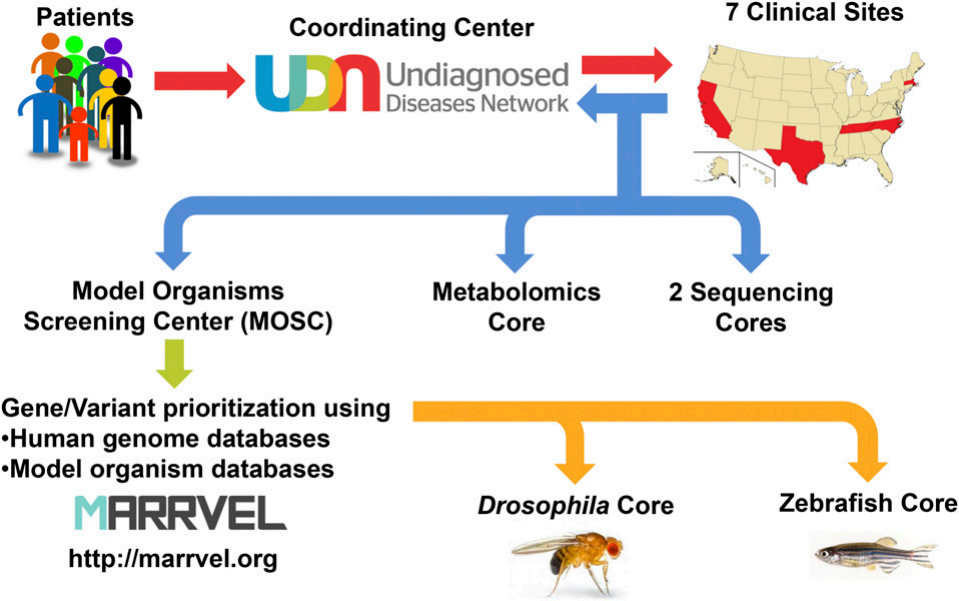
The workflow of the UDN and MOSC. Patients with undiagnosed conditions apply to the UDN primarily through a website (UDN Gateway) that is hosted by the Coordinating Center. Application forms and past medical records are then screened by a case review committee to identify cases with objective findings. Once a patient is accepted, she/he will receive a clinical workup in one of the Clinical Sites. For most cases, WES or WGS are performed on the patient and immediate relatives by one of the two Sequencing Cores. In addition, untargeted metabolomics may be performed on patient samples by the Metabolomics Core. By combining the phenotype and genotype information, some cases can be solved without further investigation. If a diagnosis is not made, the clinical site submits candidate gene/variant information to the MOSC together with a brief description of the patient’s condition. The MOSC first performs a database search using the MARRVEL tool to aggregate existing information on the human gene/variant and its model organism orthologs. In addition, matchmaking with patients in other disease cohorts are attempted through collaborations to identify other individuals with similar genotype and phenotype. Once a variant is considered to be a high priority candidate, experiments to assess gene and variant function are designed by the MOSC investigators and pursued in the *Drosophila* Core or in the Zebrafish Core.

The successes of the UDP continued after expansion to the UDN (Gahl *et al.* 2016; Ramoni *et al.* 2017). The UDN follows a similar strategy as the UDP but is decentralized and involves seven Clinical Sites [Stanford Medicine, UCLA School of Medicine, Baylor College of Medicine (BCM) with Texas Children’s Hospital, Vanderbilt University Medical Center, Duke Medicine with Columbia University Medical Center, Harvard Teaching Hospitals (Boston Children’s Hospital, Brigham and Women’s Hospital, Massachusetts General Hospital) and NIH], two Sequencing *Cores* (BCM, HudsonAlpha with Illumina), one Model Organisms Screening Center (MOSC, BCM with University of Oregon), one Metabolomics Core (Pacific Northwest National Laboratory with Oregon Health & Science University), and one Coordination Center (Harvard Medical School) (Figure 2). Recent discoveries by the UDN provided key insights into phenotypic expansion of *NR5A1* in human sex determination (Bashamboo *et al.* 2016), *ASXL2* and *Shashi-Pena syndrome* (MIM# 617190) (Shashi *et al.* 2016), and *EBF3* and *hypotoia*, *ataxia and delayed development syndrome* (MIM# 617330) (Chao *et al.* 2017a), a gene discovery that was also made simultaneously by the CMG and the Deciphering Developmental Disorders Study (DDDS) (Harms *et al.* 2017; Sleven *et al.* 2017). Use of WES, and, more recently, whole-genome sequencing (WGS) by the UDP and UDN are key in providing diagnostic results for patients who would have remained undiagnosed.

Given its approach as a network providing in-depth clinical evaluation for the most difficult to diagnose cases, the UDN faces several challenges. For example, genomic discovery in single patients is dramatically hampered by the lack of statistical power. In this context, the need for the UDN to functionally validate candidate gene variants is paramount. Therefore, extensive phenotyping (performed by the Clinical Sites and Metabolomics Core), precise genotyping (performed by the Sequencing Cores), functional exploration of candidate genes and variants (performed by the MOSC) and integration of this information (performed by the Coordination Center) are all key aspects of such a patient-centered effort.

The overall strategy, work flow and some of the key features of the UDN MOSC are highlighted in Figure 2. The MOSC receives variants submitted by the UDN Clinical Sites. Each Clinical Site integrates genotype and extensive phenotype information to prioritize candidate variants. The MOSC investigators communicate closely with the primary UDN physicians who evaluated the patient, and this dialogue continues throughout the process of investigation. The first step of the MOSC pipeline is to utilize databases to prioritize or filter candidate genomic variants. This quality control step is crucial for the MOSC to confirm that the submitted variant has a high chance of being pathogenic prior to designing and performing the actual experiments in model organisms. Public human exome and genome sequences of individuals who lack severe early-onset Mendelian disorders, such as those in the ExAC (Exome Aggregation Consortium) browser (Lek *et al.* 2016), are cross-referenced to the genes and variants from UDN patients. The rationale is that, if a similar genotype is found in people without the disease phenotype, it is unlikely that variant of interest in a candidate gene is responsible for the patient’s phenotype. However, one needs to be cautious that this filtration may not be possible for adult onset diseases if the variant in the population genomics databases are from younger individuals that may go on to develop the same disease at a later time point in life. Next, additional human databases with Mendelian disorders, including WES data from the CMG, are studied to identify candidate gene or variant matches. Using this strategy of database integration, the UDN MOSC recently aided in the diagnosis of a previously undiagnosed developmental syndrome (Schoch *et al.* 2017). From the WES data of a patient in the UDN with a sporadic phenotype of infantile epilepsy, cataracts, and developmental delay, the Duke Clinical Site identified a *de novo* missense variant in the *nucleus accumbens associated 1* (*NACC1*) gene as one of several candidates for the child’s disorder. Very little gene and protein function information was available to aid in determining whether the *NACC1* variant might be disease-causing. Working with the MOSC, a resulting collaboration between the UDN and the CMG, posting of the gene on GeneMatcher, and identification of additional cases in clinical laboratories including Baylor Genetics and UCLA Clinical Genomics Center, demonstrated a clear role for this *de novo* variant in the patient’s phenotype. The combined data of these efforts led from the initial “*n* = 1” to identification of a total of seven individuals from seven independent families, all with the same *de novo* missense variant in *NACC1* and presenting with the same disease phenotypes (Schoch *et al.* 2017). In this case, the presence of a unique variant and a unique phenotype made collaboration and matchmaking between studies and centers the key to discovery, and model organism studies were not necessary to prove the causality of this *de novo* variant. Nevertheless, this example clearly illustrates how the prescreening step prior to experimentation in model organisms can be productive. In addition, having the ability to interface with physicians and other scientists, through GeneMatcher and other matchmaking efforts, will allow model organism researchers to contribute to the diagnosis prior to the actual model organism work.

After searching for additional cases, the MOSC uses model organism databases including yeast, worm, fly, fish, and rodents to understand the extent to which the gene is conserved, where and when the gene is expressed, what the loss of function phenotypes are, whether the site of the candidate human pathogenic variant is conserved within the orthologous proteins of model organisms, and what tools and reagents have already been generated to study the gene. A recently developed web-based tool named MARRVEL (Model organism Aggregated Resources for Rare Variant ExpLoration) automates most of these searches and allows anyone to quickly retrieve the relevant information in a simple comprehensive format (Wang *et al.* 2017).

Next, genes are assigned to the *Drosophila* or Zebrafish Core facilities, where the genes and variants from the UDN patients are studied for their functional impact, to provide a better understanding of the relationships among the gene, variant, and disease phenotypes (Figure 1). Genes that are conserved in *Drosophila* (∼65% of human genes) are assigned to the *Drosophila* Core, and the remaining (∼35%) genes are assigned to the Zebrafish Core. Occasionally, variants identified in diseases that affect vertebrate specific organ systems such as bone and neural crest derived structures are also assigned to the fish core.

**Figure 3:**
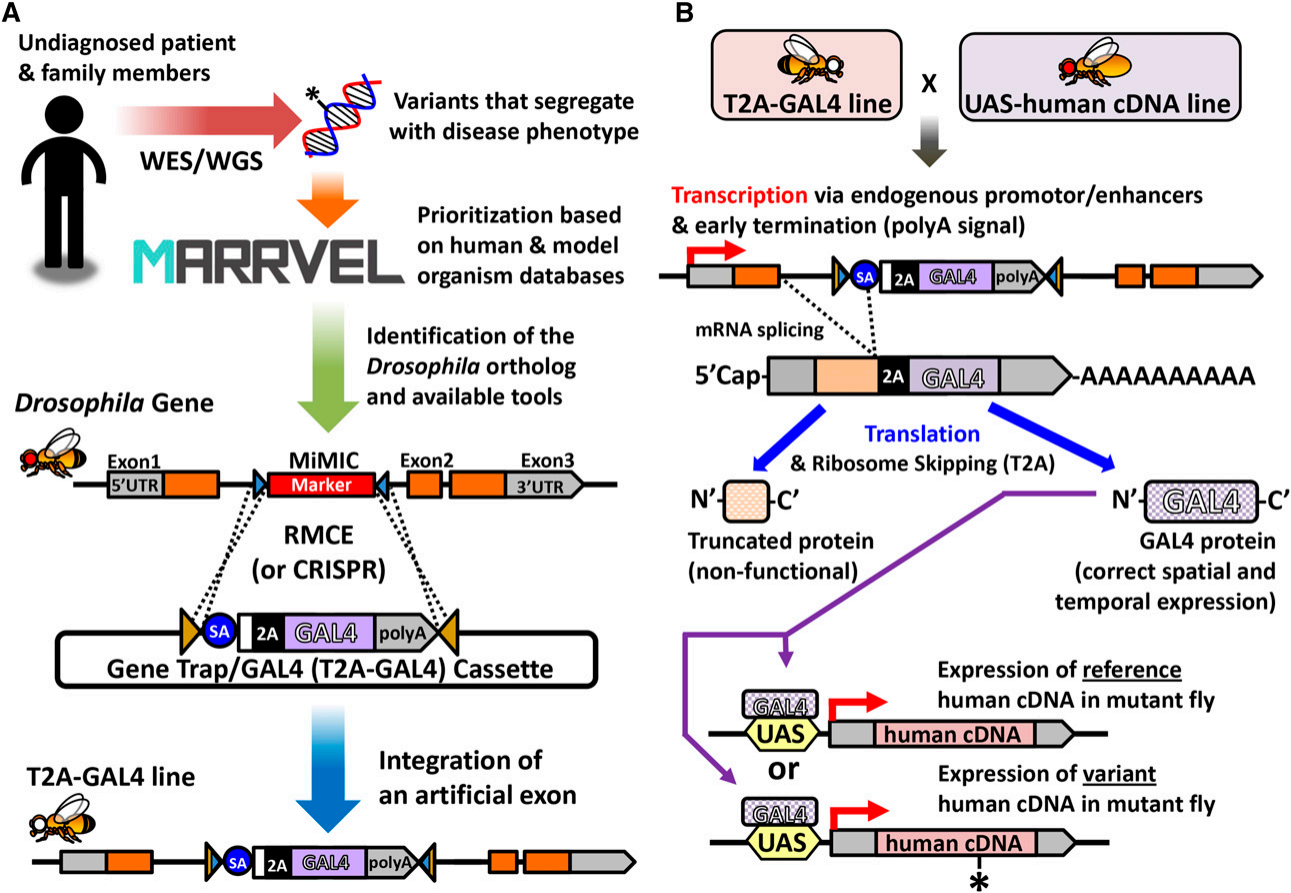
Strategy to “humanize” a *Drosophila* gene to assess functional consequences of a novel variant. (A) For most genes, the *Drosophila* Core of the MOSC performs functional studies of a patient variant by humanizing the orthologous gene in the fly. First, a fly gene that is most likely to be the ortholog of the human gene is identified using the MARRVEL tool. MARRVEL also provides a link to the FlyBase page that displays known biological function, transcriptomics and proteomics data, mutant phenotypes and available resources for the *Drosophila* gene of interest. If a coding intronic MiMIC is available, the *Drosophila* Core uses this as an entry point to study the gene. Through recombinase-mediated cassette exchange (RMCE), an artificial exon is integrated that functions as a gene trap, creating a strong LOF allele. This artificial exon contains a T2A ribosomal skipping sequence and a coding sequence for the GAL4 transcriptional activator (T2A-GAL4). (B) By crossing the T2A-GAL4 strain to a transgenic fly that carries a UAS-human cDNA construct (together with a deficiency of the locus or an independent mutant allele of the fly gene, data not shown), the fly gene can be humanized. When the gene of interest is transcribed, the splice acceptor (SA) in the artificial exon splices into the upstream exon. Since a transcription termination sequence (polyA) is present at the 3′ end of this artificial exon, the transcript is terminated, and the remaining portion of the fly gene is not transcribed. When this transcript is translated, a truncated protein that is usually nonfunctional is made together with a GAL4 protein. GAL4 is expressed in the same spatial and temporal pattern as the fly gene, allowing expression of the corresponding human cDNA under the control of the UAS element (GAL4 target sequence). By comparing the ability of the reference (wild type) and variant (mutant) to rescue the fly mutant phenotype, one can assess whether the variant of interest impacts protein function.

In the *Drosophila* Core, experiments are tailored to each gene and variant using diverse genetic strategies. One strategy depends on the versatile engineered transposable element, MiMIC (*Minos*-mediated integration cassette) (Venken *et al.* 2011), which allows “humanization” of *Drosophila* genes (Bellen and Yamamoto 2015) (Figure 3). This pipeline allows for: (1) generation of strong LOF alleles in the fly gene of interest; (2) expression of the yeast GAL4 transcription factor driven by endogenous enhancers of the gene of interest; (3) functional replacement of the fly gene with the reference (wild-type) human gene to test for rescue of the fly mutant phenotype with the orthologous reference human cDNA expressed under control of a GAL4 expressed in the proper spatial and temporal pattern; and (4) variant function analysis through expression of the human disease allele variant in the same fly line. Other strategies are also used because different reagents (*e.g.*, well characterized LOF alleles, experimentally verified RNAi lines) may be available or rescue with human cDNA may not always be successful (Harel *et al.* 2016b).

In the Zebrafish Core, CRISPR/Cas9 gene editing technology is used to target mutations into the orthologous gene and to LOF alleles in regions of the gene encoding critical functions, such as DNA binding domains, protein interaction domains, or enzymatic catalytic sites. The Zebrafish Core injects CRISPR guide RNAs (gRNAs) and Cas9 mRNA or protein into one-cell zebrafish embryos, where gene editing occurs during early cleavage divisions of the embryo. This results in large clones of mutant cells, several of which almost invariably enter the germline. To increase throughput, the Core combines multiple gRNAs targeting different genes in the same injection (Shah *et al.* 2015). This multiplexing strategy reduces the number of animals to be raised because each individual carries mutations in multiple genes. After using PCR to genotype adults developed from injected embryos, recovering mutant alleles after outcrosses, and separating mutant lines in subsequent generations, the Zebrafish Core applies a wide range of assays to characterize mutant phenotypes. Phenotypic features found in the zebrafish mutants are compared to the patients’ clinical features, and investigations to study the underlying pathobiology are initiated. Subsequent rescue experiments with wild-type and variant cDNAs or mRNAs can validate the pathogenicity of the variants.

Through these strategies, the UDN MOSC recently aided the identification of novel cases and the functional validation of *de novo* variants in *EBF3* using *Drosophila* (Chao *et al.* 2017a). Starting with a case from the UDP of a child with a neurodevelopmental disorder with a *de novo* variant of unknown significance in *EBF3* (*Early B-Cell Factor 3*), the MOSC identified two additional patients with *de novo* variants altering the same amino acid of the EBF3 protein through collaborative database searches. In addition, using a MiMIC insertion line in the fly *EBF3* ortholog *knot*—a gene that was well-studied in the context of neural development—the MOSC showed that a reference human *EBF3* cDNA can rescue the lethality of the fly *knot* LOF mutation, showing that human *EBF3* can functionally replace the *Drosophila* ortholog. The missense *de novo* variants found in the patient abolished the ability of human *EBF3* to rescue mutant flies (Chao *et al.* 2017a). Combined with *in vitro* studies, this work provided clear evidence that the *EBF3 de novo* variants are pathogenic in the three cases. Both statistical human genetic evidence and the fly functional analysis support this conclusion (Chao *et al.* 2017a). In parallel, two other groups simultaneously provided additional cases of *EBF3 de novo* variants (Harms *et al.* 2017; Sleven *et al.* 2017). This study is the first to demonstrate the effectiveness of the MOSC pipeline in variant validation for the UDN.

### The Canadian RDMM network

**Figure 4:**
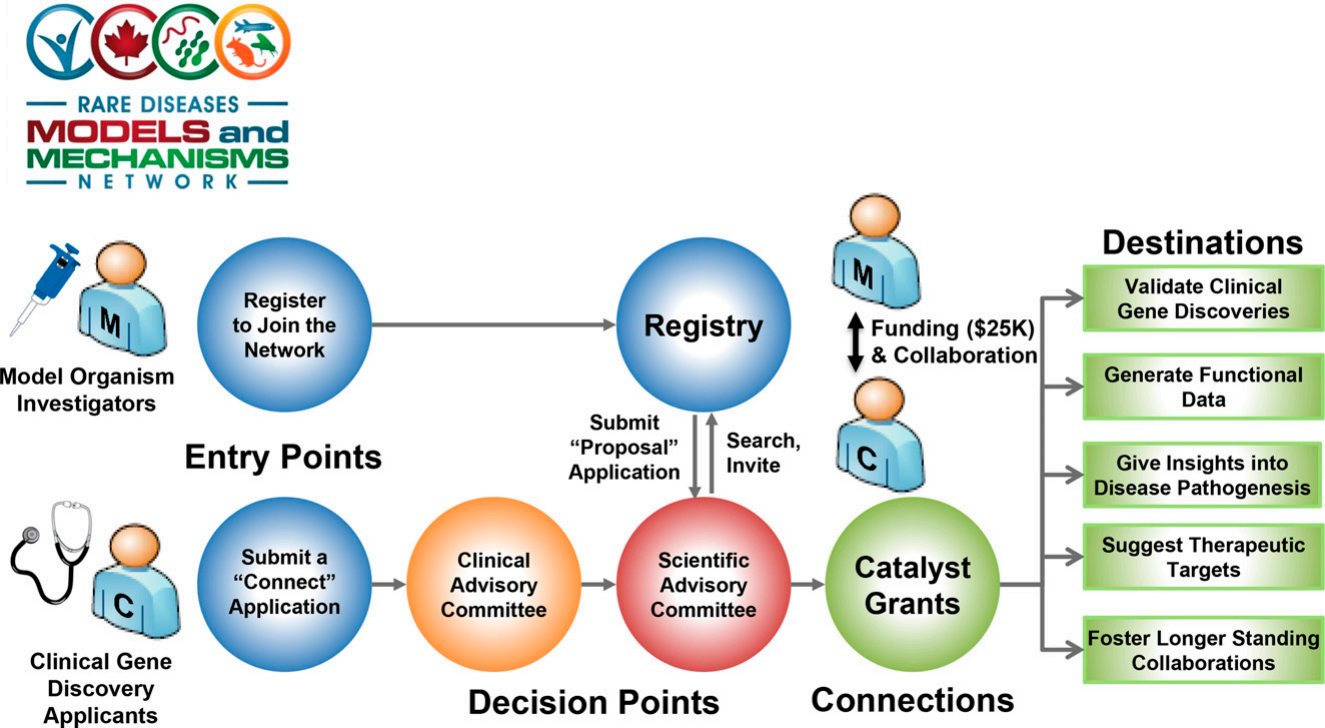
The workflow of the Canadian RDMM Network. RDMM connects Canada’s disease gene discovery projects with the Canadian model organism researchers. Investigators that work with yeast, *C. elegans*, *Drosophila*, zebrafish, or mouse are encouraged to join the network. Upon registration, the investigator provides a list of genes or genetic pathways in which they are experts. In parallel, a physician or a human geneticist submits a “connect application” for cases that they wish to find a model organism collaborator for. If the case is approved by the Clinical Advisory Committee, the Scientific Advisory Committee performs a search of the model organism registry and identifies an investigator that specializes in the orthologous gene. Upon matchmaking, the model organism investigator and the physician/human geneticist discuss a working plan and submit a proposal to the Scientific Advisory Committee. If the case is approved, funding is provided (1 year, Can$25,000) to generate a disease model and study the candidate gene/variant. The long-term goal of this process is to connect clinicians and basic researchers to establish a collaborative network across the country to facilitate rare disease research.

In Canada, a series of rare disease gene discovery projects, including FORGE (Finding of Rare Disease Genes) (Beaulieu *et al.* 2014), Care4Rare (Sawyer *et al.* 2016), IGNITE (Identifying Genes and Novel Therapeutics to Enhance Treatment), Omics2TreatID (Tarailo-Graovac *et al.* 2016), and TIDE BC (Sayson *et al.* 2015; Tarailo-Graovac *et al.* 2016), led to the identification of >300 disease genes over the past 5 years. A complementary national project was established to expedite collaboration between basic scientists and clinicians for model organism-based functional studies of rare disease gene variants, and for the development of new therapeutic strategies using model systems. The RDMM connects Canada’s disease gene discovery projects with the Canadian model organism communities of yeast, *C. elegans*, *Drosophila*, zebrafish, and mouse (Figure 4). Collaborations are established between clinicians and basic researchers as soon as possible after the initial identification of candidate genes, and a rapid response seed grant is awarded to support immediate functional experiments—a unique feature of the RDMM. The anticipated outcomes are: (1) elucidation of gene function and functional validation of human genetic variants that cause disease; (2) high impact publications of disease gene discoveries through inclusion of functional data; (3) potential rationale for treatment (*e.g.*, identification of candidate drug targets) via knowledge of a disease gene pathway; and (4) establishment of longer term collaborations between basic scientists and clinicians that will lead to basic or applied research.

Using the RDMM infrastructure, a clinician or an investigator in a genetics laboratory who identifies a new candidate gene can submit a “connect application” (Figure 4). Connect applications can also be submitted by a clinical researcher who wants to propose a model organism project for a known disease gene but for which pathophysiological understanding or treatment options are lacking. The Clinical Advisory Committee evaluates these applications within 2 weeks, and, if approved, the network registry is searched for potential orthologous gene matches that identify a model organism researcher who is an expert on the gene of interest. To generate this registry, all investigators working with model organisms in Canada were encouraged to establish an RDMM account and submit genes they designate as tier 1, 2, or 3 depending on the researcher’s level of expertise and availability of models in their laboratory. Selected investigators are invited to write a short “catalyst grant proposal application” on how the researcher would assess the functional consequences of mutations. The intention is to rapidly identify researchers who have a history and track record of studying the function of orthologs of rare disease-linked genes. Ideally, the selected individual is poised to immediately conduct functional analyses that will provide new biological insight into a human disease gene. The model organism investigator is given a year to generate a model and provide a report with the collaborating clinician. The goal of this process is not only to validate candidate genes, investigate known disease genes, and generate models to test possible drugs, but also to facilitate links between clinicians and basic researchers to create collaborative networks that will lead to larger projects.

To date, over 400 model organism researchers have registered in the database, entered over 5800 genes of interest, and 46 catalyst grants were funded since January 2015. RDMM-funded projects already led to identification of *NANS deficiency* (MIM# 610442) (van Karnebeek *et al.* 2016)—a condition with skeletal dysplasia and developmental delay caused by insufficient sialic acid synthesis. Knockdown of the orthologous zebrafish *nansa* gene, encoding sialic acid synthase, in embryos resulted in abnormal skeletal development similar to the patients’ phenotype. This phenotype in zebrafish mutants was partially rescued by the addition of sialic acid to the culture water, opening the door to potential treatment with oral sialic acid in human patients. Another example is the identification and characterization of a dominant mutation in *NALCN*, which encodes a cation channel, causing intellectual disability, ataxia, and arthrogryposis (Aoyagi *et al.* 2015)—a gene previously associated only with a recessive condition with developmental delay and hypotonia (MIM# 615419). Introduction of the analogous variant into the *C. elegans* homolog *nca-1* induced a dominant GOF neurological phenotype, providing evidence that the disease-linked variant likely has a similar impact on the human NALCN protein.

### Facilitating international collaboration through rare and undiagnosed disease research consortia

A number of institutes and organizations around the world are actively engaged in undiagnosed rare diseases research: Japan [Initiative on Rare and Undiagnosed Diseases (IRUD)]; Italy (Telethon Undiagnosed Diseases Program); and Australia and New Zealand [Undiagnosed Diseases Program—Australia and New Zealand (Baynam *et al.* 2017)]. Recent establishment of large international consortia, such as the Undiagnosed Diseases Network International (UDNI) and the International Rare Diseases Research Consortium (IRDiRC), is beginning to facilitate interactions and information exchange among undiagnosed disease researchers worldwide. Patient advocate organizations such as National Organization for Rare Disorders (NORD) and European Organization for Rare Diseases (EURORDIS) collaborate with these consortia to facilitate diagnoses and research of rare diseases. In the absence of functional data, much of the current efforts are focused around identifying a second patient with overlapping phenotypes and genotypes to try to solve the more difficult “*n* = 1” cases. Considering that functional information of genes and variants will be necessary for most new disease genes and variants of unknown significance that are discovered through these efforts, we foresee that an international network of model organism researchers will boost the pace of disease gene discovery and downstream mechanistic studies necessary for understanding pathogenesis.

## The Future of Integrating Human Genomics and Model Organisms

The drive to assess the function of human genes based on model organism research will continue to increase. As our knowledge deepens, understanding the role of human genes will greatly benefit from this knowledge base. Several general themes are emerging that will pave the way forward.

### Pleiotropy and undiagnosed disease

Pleiotropy is the phenomenon of mutations in the same gene causing multiple phenotypes. This can be due to a gene being used in different tissues or at different stages of development, or encoding a protein that has more than one molecular function. Insights from human genome sequencing efforts provided several examples of pleiotropy. For instance, sequencing often uncovers a known disease gene in a patient with undiagnosed disease, and, in retrospect, the variant is diagnostic. However, the gene might not have been considered initially because the patient did not precisely match what was previously understood about the clinical phenotype of the disease; in retrospect, the patient’s symptoms extended beyond what were previously recognized as the core clinical features. This realization of “phenotypic expansion” is a product of limited clinical descriptions and the inherent pleiotropy of many human genes (Chong *et al.* 2015). Phenotypic expansion often continues long after the gene is first associated with a disease. Pleiotropy in human disease can also manifest as a single gene associated with multiple distinct diseases. For example, pleiotropic effects are evident for the *TRPV4* locus with different mutations causing distinct diseases, as described earlier. Therefore, the key role of model organisms to facilitate understanding of the many functions of a pleiotropic gene cannot be overstated.

### Noncoding variants and model organisms

For Mendelian disease, WES is a successful clinically available diagnostic tool. However, WES only targets coding regions, and noncoding genetic variants can also play an important role in human disease (Zhang and Lupski 2015). For example, *microRNA-140* (*miR-140*) regulates palate formation in zebrafish (Eberhart *et al.* 2008), and a single nucleotide polymorphism (SNP, rs7205289) within the human *miR-140* gene, which decreases miR-140 processing, was found to be strongly associated with nonsyndromic cleft palate in human patients (Li *et al.* 2010). While some annotated noncoding but transcribed elements, such as miRNAs and long noncoding RNAs (lncRNAs) could be added to the target for sequencing, the identification of causative sequence variants in regulatory DNAs is difficult due to our incomplete understanding of the logic of human gene regulatory networks. Furthermore as additional WGS data become available, more noncoding variants will be identified. Understanding the consequences of these variants will continue to be a very significant challenge. Although there are examples of pathogenic noncoding copy number variants (CNVs) upstream of disease loci (McCarroll *et al.* 2008; Zhang *et al.* 2010), the current view is that the vast majority of disease-causing mutations from rare Mendelian disorders relate to coding or splicing variants. Interestingly, most GWAS (Genome Wide Association Studies) loci associated with common disorders are genetic variants in noncoding or regulatory regions (Edwards *et al.* 2013). Given the overall lack of conservation between noncoding regions in humans and model organisms, it is challenging to study noncoding variants, although some strategies are emerging in vertebrate model organisms (Zhang and Lupski 2015). In cases where the noncoding region is not conserved, model organism strategies to understand the consequences of changes in gene expression may offer the best insights. For example, if the variant can be shown to affect gene regulation in human cells or tissues (*e.g.*, gene expression data from RNA-seq in patient samples), then gene knockdown or overexpression in model organisms may be productive (Wangler *et al.* 2017). However, as the field elucidates more noncoding variants associated with diseases, then more direct ways to study these regulatory variants may be needed.

### Human database integration and matchmaking

Genetic diagnoses have greatly benefitted from information in numerous human databases. Efforts to use human genomics for diagnosis depend on comparing candidate variants to other variants at the locus found in other databases. The success of efforts, such as Deciphering-Developmental-Disorders-Study (2015), ExAC and its expansion project gnomAD (Genome Aggregation Database) (Lek *et al.* 2016), Geno2MP (Chong *et al.* 2016a), and the DiscovEHR Collaboration (Abul-Husn *et al.* 2016; Dewey *et al.* 2016), in creating publically available datasets is a result of these diagnostic needs. Use of integrated match-making programs that comprise the Matchmaker Exchange (MME) (Philippakis *et al.* 2015) allows the identification of patients with similar phenotypes and genotypes across different platforms. In Matchmaker Exchange, databases such as GeneMatcher (Sobreira *et al.* 2015b), PhenomeCentral (Buske *et al.* 2015), DECIPHER (DatabasE of genomiC varIation and Phenotype in Humans using Ensembl Resources) (Chatzimichali *et al.* 2015), MyGene2 (Chong *et al.* 2016b), *matchbox/seqr*, and Australian Genomics Health Alliance (AGHA) Patient Archive are connected to one another, thereby supporting queries of data from >20,000 unrelated patients, and facilitating prioritization of genes and variants.

### Model organism resources and infrastructure

Maintaining and expanding the resources and infrastructure to support model organism research will be essential for the future of both undiagnosed and diagnosed disease research, for our ability to identify molecular mechanisms quickly and efficiently, and to develop potential therapies for human disease (Table 3). We strongly feel this should be embraced and well supported by the NIH and government agencies in other countries that fund biomedical research. Indeed, rapid analyses of variants of unknown significance and deeper mechanistic studies often depend upon the availability of reagents in model organisms. If these resources are not available and need to be developed, researchers will be less likely to embark on such studies. Similarly, the organism-specific databases, including *Saccharomyces* Genome Database (SGD, budding yeast), Pombase (fission yeast), WormBase (*C. elegans*), FlyBase (*Drosophila*), The Zebrafish Information Network (ZFIN, zebrafish), Mouse Genome Informatics (MGI, mouse), and Rat Genome Database (RGD, rat), are critical to mine, organize, and curate biological information required to prioritize candidate disease genes. Although databases and mining tools integrating information from multiple key model organism databases and corresponding human genes are being developed by the Alliance of Genome Resources, the Monarch Initiative, and other efforts such as MARRVEL and Gene2Function, specific model organism databases will continue to play a critical role because they curate and annotate organism-specific datasets that are seminal to their respective research communities. For example, FlyBase (Bilder and Irvine 2017) and ZFIN (Howe *et al.* 2017) are queried tens of millions of times a year, mostly by members of the fly and zebrafish communities, respectively, which are estimated to consist of >6000 researchers each. Hence, both an integrated databases and model organism-specific databases are required to facilitate the synergy between the medical genetics and model organism research communities.

**Table 3:** Online resources cited

Comments from the NIH Leadership on Model Organism Research Support	
Francis Collins at TAGC 2016 on YouTube	https://www.youtube.com/watch?v=f9FXNU1YWQo
NIH Extramural Nexus | Open Mike | A Look at Trends in NIH’s Model Organism Research Support.	https://nexus.od.nih.gov/all/2016/07/14/a-look-at-trends-in-nihs-model-organism-research-support/
Rare and undiagnosed disease research consortia and organizations	
CMG-The Centers for Mendelian Genomics	http://mendelian.org/
DDDS-Deciphering Developmental Disorders Study	https://www.ddduk.org/
DiscovEHR collaboration	http://www.discovehrshare.com/
EURORDIS-The European Organization for Rare Diseases	http://www.eurordis.org/
IGNITE project	http://igniteproject.ca/
IRDiRC-The International Rare Diseases Research Consortium	http://www.irdirc.org/
IRUD-The Initiative on Rare and Undiagnosed Diseases	http://www.amed.go.jp/en/program/IRUD/
NORD-National Organization for Rare Disorders	https://rarediseases.org/
RDMM-The Rare Diseases: Models and Mechanisms Network	http://www.rare-diseases-catalyst-network.ca/
Telethon Undiagnosed Diseases Program	http://www.telethon.it/node/49281
UDN-The Undiagnosed Diseases Network	https://undiagnosed.hms.harvard.edu/
UDN | MOSC-Model Organisms Screening Center	https://undiagnosed.hms.harvard.edu/research/model-organisms/
UDNI-Undiagnosed Diseases Network International	http://www.udninternational.org/
Human genomic databases, tools and resources	
Australian Genomics Health Alliance Patient Archive	https://mme.australiangenomics.org.au/
ClinicalTrials.gov|Leflunomide+Vemurafenib in V600 Mutant Met. Melanoma	https://clinicaltrials.gov/ct2/show/study/NCT01611675
DECIPHER-DatabasE of genomiC varIation and Phenotype in Humans using Ensembl Resources	https://decipher.sanger.ac.uk/
ExAC (Exome Aggregation Consortium) browser	http://exac.broadinstitute.org/
GeneMatcher	https://genematcher.org/
Geno2MP	http://geno2mp.gs.washington.edu/Geno2MP/
gnomAD-Genome Aggregation Database	http://gnomad.broadinstitute.org/
* matchbox/seqr* at Broad Institute	https://seqr.broadinstitute.org/
Matchmaker Exchange	http://www.matchmakerexchange.org/
MyGene2	https://mygene2.org/MyGene2/
OMIM-Online Mendelian Inheritance in Man	https://www.omim.org/
PhenomeCentral	https://phenomecentral.org/
Cross-species mining tools and resources	
Alliance of Genome Resources	http://www.alliancegenome.org/
DIOPT-DRSC Integrative Ortholog Prediction Tool	http://www.flyrnai.org/diopt
Gene2Function	http://www.gene2function.org/
MARRVEL-Model organism Aggregated Resources for Rare Variant ExpLoration	http://marrvel.org/
Monarch Initiative	https://monarchinitiative.org/
Model organism databases, tools and resources	
BDSC-Bloomington Drosophila Stock Center	http://fly.bio.indiana.edu/
BDSC | Human proteins under the control of UAS	http://fly.bio.indiana.edu/Browse/uas/uas_hsap.php
CGC-Caenorhabditis Genetics Center	https://cgc.umn.edu/
FlyBase	http://flybase.org/
MGC-Mammalian Gene Collection	https://genecollections.nci.nih.gov/MGC/
MGI-Mouse Genome Informatics	http://www.informatics.jax.org/
MMR-Mouse Mutant Resource	https://www.jax.org/research-and-faculty/tools/mouse-mutant-resource
MMRRC-Mutant Mouse Resource and Research Centers	https://www.mmrrc.org/
Pombase	https://www.pombase.org/
RGD-Rat Genome Database	http://rgd.mcw.edu/
SGD-Saccharomyces Genome Database	http://www.yeastgenome.org/
WormBase	http://www.wormbase.org/
ZFIN-The Zebrafish Information Network	http://zfin.org/
ZIRC-Zebrafish International Resource Center	https://zebrafish.org/

In addition to these digital resources, the UDN, RDMM and similar projects in other countries will rely on publically distributed reagents and strains to facilitate gene and variant annotation. For example, a public collection of human cDNA libraries being assembled by the Mammalian Gene Collection (MGC) consortium (Temple *et al.* 2009). Transgenic yeast (Kachroo *et al.* 2015), *Drosophila*, and zebrafish (Davis *et al.* 2014) strains that allow expression of these human cDNAs *in vivo* in model organisms will provide easily accessible and valuable “off-the-shelf” resources to support and encourage the use of model organisms for functional analyses of human variants. In addition, a collection of gene knock-in (Nagarkar-Jaiswal *et al.* 2015) or BAC/phosmid transgenic lines (Sarov *et al.* 2016) that tag most proteins in the genome with epitopes or fluorescent proteins would allow investigators to assess expression, subcellular distribution, and function of genes quickly by integrating cell biological, biochemical, and proteomic approaches. For example, GFP-tagged strains can also be used to conditionally knockdown the transcript or protein of interest in a reversible and temporally and spatially controlled manner (Nagarkar-Jaiswal *et al.* 2015). Stock centers, including the Bloomington *Drosophila* Stock Center (BDSC), *Caenorhabditis* Genetics Center (CGC), Zebrafish International Resource Center (ZIRC), Mutant Mouse Resource and Research Centers (MMRRC), and Mouse Mutant Resource (MMR) that assist in the maintenance of these and other useful mutant and transgenic resources depend upon continued support by the NIH, international research community, and end-users. By combining these resources, and the genotype to phenotype information obtained from human patients, one can more rapidly study the LOF phenotype, test whether the molecular function of the human and model organism genes are conserved, determine whether a variant of unknown significance is functional, study the expression pattern and subcellular localization of the protein of interest, identify physical interaction partners, and understand the function of the gene in the organ system and cell type of interest (Bellen and Yamamoto 2015). Maintaining and expanding the resources of model organism communities will provide invaluable information to expand our ability to diagnose and dissect the molecular mechanisms underlying human diseases.

Finally, nonmodel organisms may also provide key insights into disease pathogenesis (Russell *et al.* 2017). Indeed, some animals adapted to a specific environment show a phenotype that would be lethal or detrimental in humans. These include osteopenia and profound anemia in Antarctic icefish or eye loss and metabolic syndromes in cavefish (Albertson *et al.* 2009). These organisms can be considered as “evolutionary mutant models,” which may help us better understand similar disease conditions in human. These organisms may also provide critical clues about key molecular mechanisms that could be targeted for therapeutic interventions.

### Collaboration and teamwork

Teamwork is paramount to drive model organism research in undiagnosed disease. Collaboration between clinicians who identify potential genes of interest and biologists who study model organisms to assess the function of homologous genes forms a first layer. In the CMG model, the clinicians and researchers seek collaboration based on the large number of candidate variants identified. Collaborative efforts are established with model organism researchers on a case-by-case basis. Although this strategy has been very productive, it may be difficult to expand outside large genomic centers. This justifies the need for approaches like the RDMM and UDN. In the UDN model, novel human variants are provided to a central entity, the MOSC, which is responsible for rapid assessment of the variants. The advantages are that the MOSC follows a standard approach in defined model organisms so that each variant is subject to the same analytical process. This tactic allows coordinated and systematic processing of numerous variants simultaneously. However, this process may not take full advantage of the rich expertise of investigators in particular areas. In the RDMM model variants are distributed, and the advantage of this organization is that novel variants are studied by experts with strong interest in a particular gene, tissue, or pathway. However, the methods and mechanistic depth for each variant may differ significantly because a centralized organization allows for a more efficient workflow and better resource allocation.

It is possible that a model combining the centralized (UDN-MOSC) approach and the distributive (RDMM) approach may ultimately be the best way to study candidate variants in model organisms. A potential workflow would be first to engage a primary center where genome biologists check variants against other human genomic datasets, chose the ideal model organism, select the best technologies, efficiently and economically generate the reagents, and rapidly test candidate variants. The acquired information and reagents could then be distributed to expert colleagues who wish to pursue the biology of the gene and disease in depth. Structuring multi-organism teams in the face of rapidly expanding lists of candidate variants and genes is a challenge that requires clinicians, human geneticists, bioinformaticians, and model organism investigators to work together. This collaboration allows rapid translation of knowledge obtained in one species to another and avoids duplication of effort. Integrating and acknowledging the strengths of each model organism is required to accelerate discoveries that identify pathogenic mechanisms and lead to development of novel therapeutic strategies.
