# Supplementary material for: Model Organisms Facilitate Rare Disease Diagnosis and Therapeutic Research
Source: Genetics. 2017 Aug 31;207(1):9–27. doi: 10.1534/genetics.117.203067 (PMC5586389; doi:10.1534/genetics.117.203067)
Supplement: Supplementary file 1 [file 9FileS1.pdf]

**MEMBERS OF THE UDN (163)

David R. Adams	Neil A. Hanchard	Jeanette C. Papp
Mercedes E. Alejandro	Lori H. Handley	Neil H. Parker
Patrick Allard	Matthew R. Herzog	Loren DM. Pena
Euan A. Ashley	Ingrid A. Holm	John A. Phillips III
Mahshid S. Azamian	Ellen M. Howerton	Jennifer E. Posey
Carlos A. Bacino	Howard J. Jacob	John H. Postlethwait
Ashok Balasubramanyam	Mahim Jain	Lorraine Potocki
Hayk Barseghyan	Yong-hui Jiang	Barbara N. Pusey
Alan H. Beggs	Jean M. Johnston	Rachel B. Ramoni
Hugo J. Bellen	Angela L. Jones	Amy K. Robertson
Jonathan A. Bernstein	David M. Koeller	Lance H. Rodan
Anna Bican	Isaac S. Kohane	Jill A. Rosenfeld
David P. Bick	Jennefer N. Kohler	Susan L. Samson
Camille L. Birch	Donna M. Krasnewich	Kelly Schoch
Braden E. Boone	Elizabeth L. Krieg	Molly C. Schroeder
Bret L. Bostwick	Joel B. Krier	Daryl A. Scott
Lauren C. Briere	Jennifer E. Kyle	Prashant Sharma
Donna M. Brown	Seema R. Lalani	Vandana Shashi
Matthew Brush	C. Christopher Lau	Edwin K. Silverman
Elizabeth A. Burke	Jozef Lazar	Janet S. Sinsheimer
Lindsay C. Burrage	Brendan H. Lee	Ariane G. Soldatos
Shan Chen	Hane Lee	Rebecca C. Spillmann
Gary D. Clark	Shawn E. Levy	Kimberly Splinter
Joy D. Cogan	Richard A. Lewis	Joan M. Stoler
Cynthia M. Cooper	Sharyn A. Lincoln	Nicholas Stong
William J. Craigen	Allen Lipson	Kimberly A. Strong
Mariska Davids	Sandra K. Loo	Jennifer A. Sullivan
Jyoti G. Dayal	Joseph Loscalzo	David A. Sweetser
Esteban C. Dell'Angelica	Richard L. Maas	Cynthia J. Tifft
Shweta U. Dhar	Ellen F. Macnamara	Camilo Toro
Ani Dillon	Calum A. MacRae	Alyssa A. Tran
Katrina M. Dipple	Valerie V. Maduro	Tiina K. Urv
Laurel A. Donnell-Fink	May Christine V. Malicdan	Zaheer M. Valivullah
Naghmeh Dorrani	Laura A. Mamounas	Eric Vilain
Daniel C. Dorset	Teri A. Manolio	Tiphanie P. Vogel
Emilie D. Douine	Thomas C. Markello	Daryl M. Waggott

David D. Draper	Julian A. Martínez-Agosto	Colleen E. Wahl
Annika M. Dries	Paul Mazur	Nicole M. Walley
David J. Eckstein	Allyn McConkie-Rosell	Chris A. Walsh
Lisa T. Emrick	Alexa T. McCray	Michael F. Wangler
Christine M. Eng	Thomas O. Metz	Patricia A. Ward
Ascia Eskin	Matthew Might	Katrina M. Waters
Cecilia Esteves	Paolo M. Moretti	Bobbie-Jo M. Webb-Robertson
Tyra Estwick	John J. Mulvihill	Monte Westerfield
Paul G. Fisher	Jennifer L. Murphy	Matthew T. Wheeler
Brent L. Fogel	Donna M. Muzny	Anastasia L. Wise
William A. Gahl	Michele E. Nehrebecky	Lynne A. Wolfe
Rena A. Godfrey	Stan F. Nelson	Elizabeth A. Worthey
David B. Goldstein	J. Scott Newberry	Shinya Yamamoto
Sarah E. Gould	John H. Newman	Yaping Yang
Jean-Philippe F. Gourdine	Sarah K. Nicholas	Guoyun Yu
Catherine A. Groden	Donna Novacic	Allison Zheng
Andrea L. Gropman	Jordan S. Orange	Patricia A. Zornio
Melissa Haendel	J. Carl Pallais	
Rizwan Hamid	Christina GS. Palmer	
